# Applying Benefit-Cost Analysis to Air Pollution Control in the Indian Power Sector

**DOI:** 10.1017/bca.2018.27

**Published:** 2019-12-27

**Authors:** Maureen L. Cropper, Sarath Guttikunda, Puja Jawahar, Zachary Lazri, Kabir Malik, Xiao-Peng Song, Xinlu Yao

**Affiliations:** 1 Maureen L. Cropper, Department of Economics, University of Maryland, College Park, Maryland 20742, USA and Resources for the Future, Washington, D.C. 20036, USA; 2 Sarath Guttikunda: UrbanEmissions.Info, New Delhi, India; 3 Puja Jawahar: UrbanEmissions.Info, New Delhi, India; 4 Zachary Lazri: Department of Economics, University of Maryland, College Park, Maryland 20742, USA; 5 Kabir Malik: World Bank, Washington, D.C. 20433, USA; 6 Xiao-Peng Song: Department of Geographical Sciences, University of Maryland, College Park, Maryland 20742, USA; 7 Xinlu Yao: Department of Economics, University of Maryland, College Park, Maryland 20742, USA

**Keywords:** air pollution control, environment, health, health impacts of coal, Indian electricity sector, Q01, Q51, Q53

## Abstract

Air pollution is a persistent and well-established public health problem in India: emissions from coal-fired power plants have been associated with over 80,000 premature deaths in 2015. Premature deaths could rise by four to five times this number by 2050 without additional pollution controls. We site a model 500 MW coal-fired electricity generating unit at eight locations in India and examine the benefits and costs of retrofitting the plant with a flue-gas desulfurization unit to reduce sulfur dioxide emissions. We quantify the mortality benefits associated with the reduction in sulfates (fine particles) and value these benefits using estimates of the value per statistical life transferred to India from high income countries. The net benefits of scrubbing vary widely by location, reflecting differences in the size of the exposed population. They are highest at locations in the densely populated north of India, which are also among the poorest states in the country.

## 1 Introduction

In addition to contributing to climate change, air pollution from coal-fired power plants can cause significant health problems for local and regional populations if emissions are not controlled. This has led many countries to regulate plant emissions. These include emissions of particulate matter (fine particles or PM_2.5_), sulfur dioxide (SO_2_) and nitrogen oxides (NO*_x_*). Particulate matter has been linked to heart and lung disease. Emissions of SO_2_ and NO*_x_*, when they combine with ammonia in the atmosphere, also form fine particles and thus have deleterious health effects. It is estimated that emissions from coal-fired power plants caused over 80,000 premature deaths in India in 2015, and will cause over 400,000 premature deaths in 2050 if coal-fired generating capacity continues to expand (GBD MAPS, 2018).^1^

In December 2015 India issued its first regulations governing emissions of SO_2_ and NO*_x_* from coal-fired power plants and strengthened its regulations governing emissions of particulate matter from thermal power plants (Ministry of Environment, Forests, and Climate Change, 2015). PM can be controlled by washing coal to reduce its ash content (Cropper et al., 2012) or through the use of electrostatic precipitators, which have been required on coal-fired power plants in India since 1984 (Cropper et al., 2017). NO*_x_* can be controlled by using low-NO*_x_* burners and/or selective catalytic reduction (Fowlie, 2010). SO_2_ can be controlled by burning low-sulfur coal and by installing flue-gas desulfurization units.

This paper examines the costs and benefits of retrofitting coal-fired power plants in India with flue-gas desulfurization units (FGDs or scrubbers) to reduce SO_2_.^2^ Although Indian coal has low-sulfur content, meeting new emission standards will require retrofitting most coal-fired power plants with FGDs (Cropper et al., 2017). Cropper et al. (2017) examine the costs and health benefits of retrofitting the 2009 stock of coal-fired power plants in India with scrubbers, focusing on the mortality reductions that FGDs will achieve.^3^ They calculate the cost per death avoided of retrofits and estimate that retrofitting the 2009 stock of plants with scrubbers would avoid 13,000 premature deaths per year, at an average cost of $131,000 (2013$) per death avoided. They conclude that, in the aggregate, installing scrubbers results in benefits that exceed costs.^4^ They note, however, that there is considerable heterogeneity in the cost per death avoided across plants.

In this paper we conduct a benefit-cost analysis of installing an FGD on a model power plant (a 500 MW electricity generating unit) sited at eight different locations in India. Although FGDs are effectively required on all 500 MW units,^5^ quantifying the net benefits of scrubbing at individual plants can aid in prioritizing retrofitting coal-fired power plants with FGDs.^6^ We quantify the health benefits associated with the FGD, which vary significantly with the size of the population exposed to the plant’s emissions and meteorological conditions and, hence, with location. The costs of installing and operating the scrubber also vary with location. We assume that wet limestone FGDs will be installed in inland areas. In coastal areas, we assume that a seawater FGD will be installed, which is cheaper to build and operate. Analyzing the benefits and cost of an FGD at a model plant allows us to focus on the impact of plant location on net benefits, holding plant size constant. Because the impact of plant emissions of SO_2_ on ambient air quality can be approximated by a linear function for small changes in emissions (European Consortium for Modelling of Air Pollution and Climate Strategies, 2012), benefits can be scaled up as a function of plant size, as can costs. We also elucidate the impact of key parameters on net benefits.

## 2 Framing of the problem

**Figure 1:**
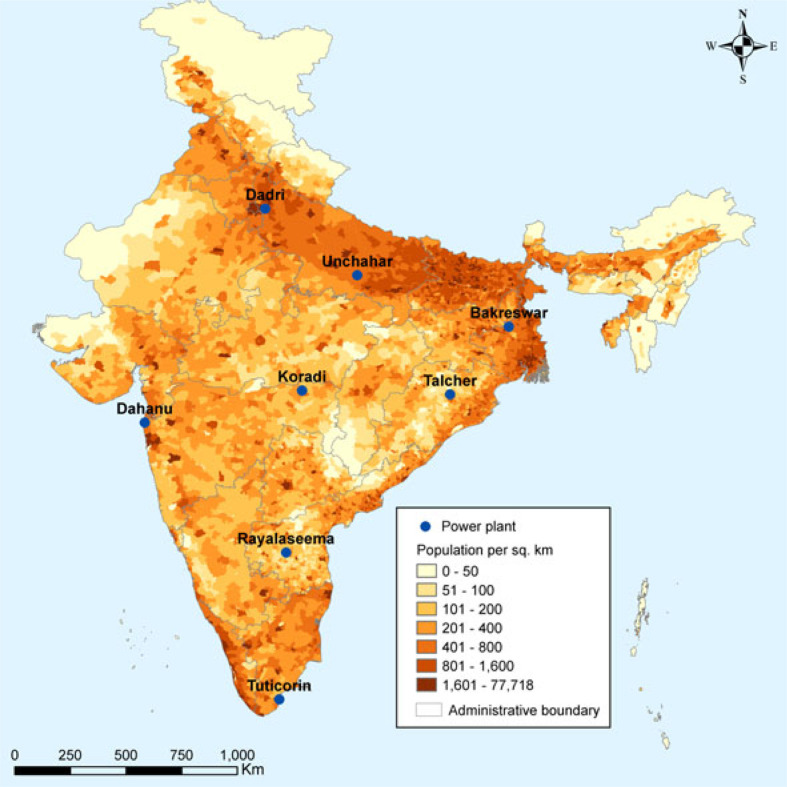
Locations of Model Power Plants.

We examine the costs and benefits of retrofitting a 500 MW coal-fired generating unit with a scrubber at the eight locations pictured in Figure 1. Coal-fired power plants currently exist at these locations, but the unit we examine is a model unit with identical operating conditions and emissions regardless of location. The eight locations vary in the impact of emitting a ton of SO_2_ on population-weighted PM_2.5_ and associated premature mortality, as described more fully below.

Analyzing the benefits of installing a scrubber requires estimating the damages associated with the plant’s SO_2_ emissions with and without an FGD. Estimating the health damages associated with SO_2_ emissions involves four steps: (1) estimating the plant’s SO_2_ emissions; (2) translating emissions into their impact on ambient air pollution; (3) estimating the effect of the plant’s impact on air pollution on human health. These steps are implemented without the scrubber, and then with the scrubber. The reduction in health impacts constitutes the health benefits of scrubbing and can be monetized (step 4), to be compared with the costs of scrubbing.

To estimate SO_2_ emissions from our model unit, we assume that the unit operates at a capacity factor of 85 percent, burns coal containing 0.5 percent sulfur by weight and consumes 0.69 kg of coal per kWh of electricity generated (Cropper et al., 2017). The 85 percent capacity factor reflects the Central Electricity Authority of India’s benchmark operating conditions. Domestic coal is approximately 0.5 percent sulfur by weight (Lu et al., 2013; CSTEP, 2018).^7^ Our assumptions imply that, without an FGD, the unit generates 20,442 tons of SO_2_ per year.

SO_2_ emissions react in the atmosphere with ammonia to form fine particles (PM_2.5_, in the form of ammonium sulfate). Because of the health impacts of PM_2.5_, we focus on this measure of ambient air quality. Translating the plant’s emissions into impacts on ambient air quality requires dispersion models that track the transport of pollutants through the atmosphere using information on weather, topography and pollutant chemistry. Dispersion models may be either process-based or reduced form (NRC, 2010). Process-based models (e.g., CAMx, CMAQ) use detailed atmospheric chemistry to simulate interactions among pollutants and gases in the atmosphere and thus account for nonlinearity in the dispersion process. Process-based models are, however, more computationally intensive than reduced-form models. Reduced-form models (e.g., CALPUFF, HYSPLIT) use simplified dispersion calculations to predict concentration changes. In this paper we use CAMx,^8^ which captures the transformation of SO_2_ into sulfate particles (PM_2.5_).^9^

We ran CAMx for 72 power plant locations in India. The model was run separately for each location, simulating 365 days of emissions, to calculate the increase in annual average fine particle concentrations corresponding to the model unit’s emissions.^10^ We assumed a stack height of 210 meters, the minimum stack height required by law for a 500 MW generating unit. The model was run at a 0.25° grid resolution (approximately 27 km×27 km) and combined with 2011 population data to calculate the population-weighted increase in annual average PM_2.5_ concentrations associated with the model plant.^11^

The eight locations chosen for our analysis are based on estimates of mortality due to fine particles associated with the plant’s SO_2_ emissions (step 3 of the analysis). Epidemiological research has found consistent associations between premature mortality and PM_2.5_. Pope et al. (2002) report significant impacts of exposure to PM_2.5_ in cites in the US on all-cause, cardiopulmonary and lung cancer mortality. This work formed the basis of early studies of the global burden of air pollution (Cohen et al., 2004). More recent studies of the Global Burden of Disease (Lim et al., 2012; GBD 2015 Risk Factor Collaborators, 2016) use meta-analyses of epidemiological studies from several sources to quantify the impact of a wider range of PM_2.5_ exposures on cardiovascular and respiratory deaths, as well as premature deaths from lung cancer and acute lower respiratory infection (ALRI) (Burnett et al., 2014).

We calculate premature mortality associated with the increase in annual average PM_2.5_ concentrations for each of 72 locations as the product of baseline deaths, by cause, and the fraction of premature deaths attributable to sulfates. The fraction of premature deaths attributable to sulfates for each disease is given by 1 − exp*(β*^∗^ *C)*, where *β* is the change in the relative risk attributable to a one microgram per cubic meter change in PM_2.5_ and *C* is the population-weighted change in ambient PM_2.5_ concentrations associated with SO_2_ emissions from the plant. The *β* coefficients were calculated using the Integrated Exposure Response functions (IERs) for ischemic heart disease, stroke, lung cancer, chronic obstructive pulmonary disease (COPD), and ALRI developed by Burnett et al. (2014) and reported by the Institute for Health Metrics and Evaluation (IHME).^12^ For each disease, the change in relative risk (*β*) was evaluated at the population-weighted annual average exposures for India used in the 2010 Global Burden of Disease (Brauer et al., 2012).^13^ Baseline deaths by age and cause were obtained from the IHME.^14^

We ranked the 72 locations by premature deaths per ton of SO_2_ emitted and selected eight of the locations to conduct the present analysis. Plant locations were chosen to represent a range of SO_2_ impacts as well as geographic locations throughout the country (see Figure 1). Table 1 lists the eight plant locations and the premature deaths per ton of SO_2_ emitted. Table 1 also lists estimates of the annual premature deaths associated with SO_2_ emissions from the model 500 MW electricity generating unit installed at each location. Two locations, Dahanu and Tuticorin, are on the coast and can therefore accommodate a seawater FGD. Baseline premature deaths range from 18 to over 500 per year. Deaths are highly correlated with population density (see Figure 1), but also reflect meteorological conditions. Premature deaths per ton are highest at the three plant locations in the north of India: Dadri, Unchahar and Bakreswar.

**Table 1:** Locations and impacts of model plants.

Plant name	State name	Annual Deaths/1000 tons SO2	Annual Deaths due to SO2
Dadri	Uttar Pradesh	24.8	507
Unchahar	Uttar Pradesh	16.3	333
Bakreswar	West Bengal	7.43	152
Dahanu	Maharashtra	4.13	84.4
Talcher	Orissa	3.19	65.1
Koradi	Maharashtra	2.19	44.8
Rayalaseema	Andhra Pradesh	1.20	24.6
Tuticorin	Tamil Nadu	0.884	18.1

## 3 Benefits analysis

SO_2_ emissions react in the atmosphere with ammonia to form fine particles (PM_2.5_, in the form of ammonium sulfate) and with water to form sulfuric acid (acid rain). Particulate matter has been linked to heart and lung disease, as described above, but also to impacts on brain development and functioning (Brockmeyer & D’Angiulli, 2016; Landrigan et al., 2017). It also impairs visibility. Acidic deposition can reduce soil quality, impair timber growth and harm freshwater ecosystems (US EPA, 2011). In estimating the benefits of reducing SO_2_ emissions, we focus on health benefits, specifically, on the mortality benefits estimated using the IERs described above.^15^ We do not quantify either the benefits of reduced morbidity associated with fine particles or the benefits of reduced acid deposition; hence our benefit estimates significantly underestimate the benefits of scrubbing SO_2_.

To calculate premature deaths avoided, we assume that installation of a scrubber will remove 90 percent of sulfur dioxide emissions.^16^ Due to the linearity of sulfate formation and the approximate linearity of concentration-response for a small change in concentrations, this reduction in emissions implies a 90 percent reduction in premature deaths attributable to SO_2_ emissions. An important question is the period over which this reduction would occur. Apte et al. (2015) assume no lag between emissions reductions and the associated reduction in premature deaths. The US Environmental Protection Agency (EPA) (2011) assumes that 80 percent of the reduction in PM_2.5_ mortality is achieved within five years of the reduction in emissions, although alternate lag structures have been considered (EPA, 2013). A conservative approach is to assume that the scrubber reduces 72 percent of premature deaths in each year of its 20-year operation. This is equivalent to assuming a lag structure that reduces the present value of premature deaths avoided to 0.8 times total premature deaths avoided.^17^ We calculate premature deaths avoided in the base year *(*Deaths Avoided_0_*)* as 0.72 (= 0.9 × 0.8) of the baseline deaths attributable to the model plant (see Table 1). We assume that premature deaths avoided increase in proportion to the projected rate of population increase (*g_l_*) which we assume to equal 0.94 percent per year (Population Reference Bureau, 2007).

Ideally, we would like to value premature deaths avoided using studies of mortality risk reductions conducted in India. The value per statistical life (VSL) reported in these studies, however, varies widely. Bhattacharya et al. (2007) report a preferred VSL estimate of Rs. 1.3 million (2006 Rs.) based on a stated preference study of Delhi residents. Madheswaran (2007) estimates the VSL based on a compensating wage study of workers in Calcutta and Mumbai to be approximately Rs. 15 million. Shanmugam (2001) reports a much higher value (Rs. 56 million) using 1990 data on workers in Madras.

We therefore value each death avoided using values transferred from high income countries, following reference case protocols developed by Robinson et al. (2018). The three VSL values, in 2015 International (PPP) dollars, are $969,600 (160 times per capita GNI), $606,000 (100 times per capita GNI) and $318,287 (53 times per capita GNI, extrapolated from the US VSL using an elasticity of 1.5). In the text we present all costs and benefits in USD at market exchange rates.^18^ The corresponding transferred VSLs in 2015$ are $256,000, $160,000 and $84,036.

We assume that the transferred VSL in the base year, VSL_0_, grows at a rate that depends on the rate of growth in per capita income in India (*g_p_*), and the income elasticity of the VSL (*ε*). Specifically, VSL*_t_* = VSL_0_[*(*1 + *g_p_)^t^*]*^ε^*. We estimate *g_p_* to be 6.3 percent per year.^19^ We use an *ε* = 1 when the VSL is transferred using an income elasticity of 1 (i.e., using the first two transfer approaches), and *ε* = 1.5 when the VSL is transferred from the US using an income elasticity of 1.5. The present discounted value of the mortality benefits from scrubbing is given by equation (1), where *r* is the rate at which benefits are discounted. We use the reference case value of *r* = 0.03 (Wilkinson et al., 2016), but also use a higher rate of *r* = 0.08. The latter is consistent with the rate of return on government bonds in India, and also reflects the higher rate of income growth in the country. As a sensitivity analysis we use a value of *r* = 0.12, or two times the near-term growth rate (motivated by the Ramsey rule).

$${\text{PDV}}_{B}=\overset{n}{\underset{t=0}{\Sigma }}{\text{Deaths Avoided}}_{0}{\left(1+g_{l}\right)}^{t}{\text{VSL}}_{0}{\left(1+g_{p}\right)}^{\epsilon t}{\left(1+r\right)}^{-t}.$$

Table 2 shows the presented discounted value of mortality benefits, computed using alternate values of VSL_0_ and alternate discount rates. The present value of benefits ranges from $3.04 billion (2015$) to $108 million using the highest VSL and *r* = 0.03. Using the conservative value of $84,036 and *r* = 0.03, they range from $1.44 billion to $51.2 million. With a VSL_0_ of $256,000, raising the discount rate from 3 percent to 8 percent lowers the present value of benefits by 40 percent; raising it to 12 percent lowers the present value of benefits by 57 percent.

**Table 2:** Present value of mortality benefits of an FGD in millions (2015$).

Plant name	VSL	$84,036	$160,000	$256,000	$256,000	$256,000
	r	3%	3%	3%	8%	12%
Dadri		1440	1900	3040	1840	1320
Unchahar		943	1250	1990	1210	866
Bakreswar		430	568	909	549	395
Dahanu		239	316	505	305	219
Talcher		184	244	390	236	169
Koradi		127	168	268	162	116
Rayalaseema		69.6	92.0	147	89.0	64.0
Tuticorin		51.2	67.7	108	65.4	47.0

An alternate approach, which we do not follow, is to use the value per statistical life year (VSLY) multiplied by the expected number of life years gained. The VSLY approach is widely used in Europe, although its theoretical foundations have been criticized (Hammitt, 2007). On average, each expected death averted leads to a gain in 25.5 life years when a scrubber is installed. If calculated using the recommendations in Robinson et al. (2018), the VSLY is estimated by dividing the VSL by the remaining life expectancy of an adult of average age. The remaining life expectancy of a 44-year old adult in India is approximately 34.4 years.^20^ Multiplying the average number of life years saved by the VSLY would, therefore, reduce the present value of mortality benefits in Table 2 by about 25 percent.

## 4 Analysis of costs

An FGD is an end-of-pipe technology that removes SO_2_ from combustion gases before they exit the smokestack. Flue gases are treated with an alkaline substance that reacts with the acidic SO_2_ to form a by-product that is removed before flue gases are emitted. In a wet limestone FGD (wFGD), gases are treated with limestone slurry, which is sprayed on the gas in an absorber unit. Gypsum, which can be sold commercially, is produced as a by-product. Another rapidly expanding technology is seawater FGDs (swFGDs). These units use the alkalinity of seawater to remove SO_2_ from the flue gases. The by-product is water, which is treated and discharged back into the sea. FGDs are capable of reducing SO_2_ emissions up to 95 percent, depending on the technology used.^21^

Both scrubber technologies are in use in India. The Indian Supreme Court required the installation of an FGD at the Dahanu plant in Maharashtra. FGDs are also in operation at the Trombay and Udupi plants. FGDs have also been commissioned at several other plants, including Dadri and Koradi.^22^ Both Dahanu and Trombay have swFGDs. Seawater FGDs have lower capital and variable costs than wet limestone FGDs, but they can be installed only in coastal areas. We assume that all FGDs installed at plants in coastal areas are swFGDs and that wet limestone FGDs are installed at all other locations.

The costs of FGD adoption include the capital costs of FGD installation and annual operating costs. The capital costs of installation include one-time equipment purchase and the costs of setting up the FGD unit and connecting it to the boiler and flue stack. Based on the type of FGD, additional equipment, such as a limestone storage unit, mill and gypsum handling unit in the case of a wet limestone FGD, or water treatment in the case of a sea water FGD, also needs to be purchased. Operating costs include periodic maintenance and labor to operate and maintain the FGD and accompanying equipment, the purchase of reagent (limestone in the case of wFGD) and by-product handling and disposal. Auxiliary consumption of electricity by the FGD is also a part of the variable costs of operation.

Table 3 shows the assumptions used to construct the cost estimates. Capital costs for a wFGD are based on costs for installing FGDs on each of two 490 MW units proposed for the Dadri plant.^23^ Capital costs for a swFGD are based on the FGD installed on two 250 MW units at the Dahanu plant (MERC, 2009). These yield a capital cost of US$93,800/MW (2015$) for a wFGD and a cost of US$72,500/MW (2015$) for a swFGD. The greater costs for a limestone FGD reflect the expenditure on reagent handling and by-product disposal facilities. In contrast, a swFGD discharges the water back into the sea and does not require as much capital investment. We increase both figures by 30 percent to reflect additional retrofitting costs. The operating and maintenance costs are based on those at the Dahanu plant (MERC, 2009). We assume that these are 30 percent higher for a wFGD (Malik, 2013).^24^

**Table 3:** Operating characteristics for cost calculations: baseline assumptions.

		Benchmark	
Capacity utilization (%)		85	
Capital discount rate (%)		3%	
Plant life (retrofit) (yrs)		20	
		FGD Type	
	Wet limestone		Seawater
Capital costs (US$/MW)	$93,800		$72,500
Fixed operating costs (US$/MWh)	$0.391		$0.297
Electricity costs (US$/kWh)	$0.0547		$0.0547
Auxiliary consumption (%)	1.50		1.25
Annual FGD electricity cost for a 500 MW unit	$3,050,000		$2,550,000
PDV of costs for a retrofitted FGD	$165,000,000		$132,000,000
PDV of costs for a new FGD	$151,000,000		$121,000,000

The present value of the cost of retrofitting a scrubber with a life of 20 years is given by equation (2), where *d* represents the rate of increase in the costs of operating the scrubber.

$${\text{PDV}}_{C}=\text{capital cost}+\overset{n}{\underset{t=0}{\Sigma }}{\left(1+r\right)}^{-t}{\left(1+d\right)}^{t}\left(O\&M+\text{electricity cost}\right).$$

Since the FGD operation and maintenance cost information for plants in India is limited, we assume that the operation and maintenance costs in India grow at the same rate as in the U.S.^25^ Using the average annual operation and maintenance cost information from the U.S. Energy Information Administration (2016), the value of *d* was determined by calculating the compound rate of growth of the operation and maintenance cost from 2005 to 2015. This value was calculated to be 4 percent. Using a discount rate of *r* = 0.03 yields a present value of $165 million (2015$) for the costs of retrofitting a wFGD and $132 million (2015$) for a swFGD. The present value of costs falls to $127 million (wFGD) and $101 million (swFGD) using *r* = 0.08.

## 5 The net benefits of retrofitting an FGD

Table 4 shows the net mortality benefits from retrofitting a model power plant at eight locations in Figure 1. We assume that a swFGD is installed at the Dahanu and Tuticorin locations and a wFGD at the other six locations. Recall that the benefits computed here are a lower bound – they reflect the value of avoided mortality only and ignore the morbidity, amenity and ecosystem benefits provided by reducing SO_2_ emissions. Using a VSL_0_ equal to 100 times per capita income ($160,000), the scrubber passes the benefit-cost test based solely on mortality benefits at all but the two least-densely populated locations. At a VSL_0_ of $84,036 (53 times per capita income), the scrubber passes the benefit-cost test at five locations, based solely on mortality benefits.

**Table 4:** Net benefits from scrubbing in millions (2015$).

Plant name	VSL	$84,036	$160,000	$256,000	$256,000	$256,000
	r	3%	3%	3%	8%	12%
Dadri		1270	1730	2870	1710	1210
Unchahar		777	1080	1830	1080	755
Bakreswar		264	403	744	422	284
Dahanu		107	184	373	204	132
Talcher		19	78.3	225	108	58.6
Koradi		–38.6	2.25	103	34.6	5.75
Rayalaseema		–95.7	–73.3	–18	–38.5	–46.7
Tuticorin		–80.6	–64.2	–23.6	–35.7	–40.5

Whether an FGD retrofit passes the benefit-cost test based solely on health benefits depends on the VSL used and also on the magnitude of morbidity and other benefits, which we do not quantify. Studies suggest that the morbidity costs of pollution-related disease may conservatively increase health costs by 10–70% (Narain & Sall, 2016; Hunt et al., 2016). Individual country studies have found morbidity costs as a percent of mortality costs to be: 25% for Colombia (Golub et al., 2014), 22–78% for China (World Bank, 2007) and 78% for Nicaragua (Kemper, 2013). Thus the numbers in Table 4 should not be interpreted as determining whether a scrubber yields net benefits at each of the eight locations. We do, however, believe that morbidity benefits will be proportional to reductions in population-weighted concentrations of PM_2.5_ and, hence, that the numbers in Table 4 can serve as a guide to prioritizing the installation of FGDs (i.e., they provide an accurate ranking of eight sites based on health benefits).

**Figure 2:**
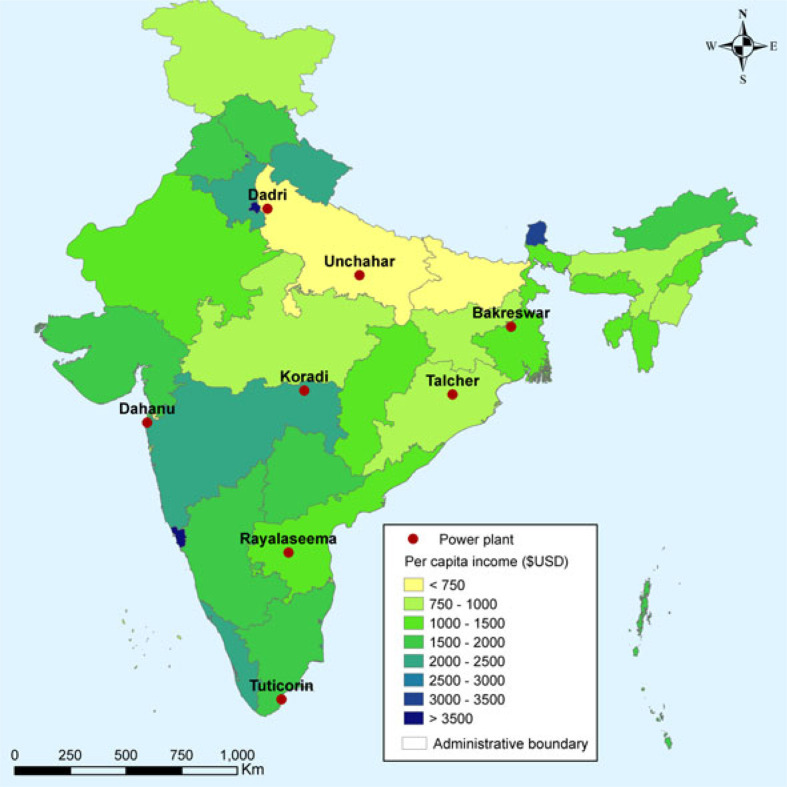
Indian States and Union Territories by GDP per Capita (2017$).

The net benefits of retrofitting the model unit based on avoided mortality are clearly greatest in the densely populated north of India. These are also some of the poorest areas in India. Figure 2 shows eight locations studied on a map of per capita GDP by state, measured for the 2014–15 fiscal year.^26^ The Dadri and Unchahar plants are located in Uttar Pradesh, one of the poorest states in India, and most of the population exposed to the Bakreswar plant lives in Bihar – the poorest state in India – as well as Jharkhand and West Bengal.

Because the VSL used in this analysis has been transferred to India based on national per capita income, it is natural to ask whether the VSL should also vary with the per capita income of the states in which benefits are received. Such an approach would better reflect the ability of the population to allocate their resources between achieving these risk reductions and paying for other goods and services. However, we follow the practice in OECD counties of applying the same VSL to all persons within the same country; hence we do not allow the VSL to vary by state.

Because benefits and costs in our analysis are well-defined, we also present benefit-cost ratios (Table 5). These results mirror Table 4 – benefit-cost ratios exceed one when net benefits are positive – but are easier to comprehend and may aid in prioritizing locations where installing FGDs would yield the greatest net benefits. We note, however, that a benefit-cost ratio below one should not be interpreted as indicating that scrubbing does not yield net benefits: our benefits estimates exclude the value of avoided morbidity, as well as impacts on ecosystems and the aesthetic value of clean air. The benefit-cost ratio and the magnitude of net benefits are, however, likely to provide a useful ranking of locations in terms of net health benefits. Morbidity benefits will increase in proportion to the reduction in population-weighted PM_2.5_ concentrations and in proportion to the size of the exposed population, which are positively correlated with mortality benefits.

**Table 5:** Benefit/cost ratios for FGD retrofits.

Plant name	VSL	$84,036	$160,000	$256,000	$256,000	$256,000
	r	3%	3%	3%	8%	12%
Dadri		8.7	11	18	14	12
Unchahar		5.7	7.5	12	9.5	7.8
Bakreswar		2.6	3.4	5.5	4.3	3.6
Dahanu		1.5	2.4	3.8	3.0	2.5
Talcher		1.1	1.5	2.4	1.9	1.5
Koradi		0.77	1.0	1.6	1.3	1.1
Rayalaseema		0.42	0.56	0.89	0.70	0.58
Tuticorin		0.31	0.51	0.82	0.65	0.54

## 6 Conclusion

This paper has two objectives. One is to demonstrate the feasibility of conducting a benefit-cost analysis of air pollution control measures in a low- or middle-income country (LMIC) setting and to illustrate the sensitivity of the results to key parameters. The second is to provide information that could help to prioritize the installation of scrubbers at coal-fired power plants in India.

Estimating the benefits of a project that reduces stationary source air emissions requires (1) calculating emissions before and after the project, (2) translating the change in emissions into a change in geo-referenced ambient concentrations, (3) estimating impacts on health and other endpoints using concentration-response functions and (4) valuing these endpoints. There are uncertainties in each step, some of which we have dealt with through sensitivity analysis and others of which we have ignored. Regarding the first step, our results should be viewed as conditional on annual SO_2_ emissions of 20,000 tons. Given the approximate linearity of results in steps 2 and 3 (for small changes in emissions), the results can be scaled according to differences in emission estimates.

It is more difficult to assess the accuracy of our projections of the impact of SO_2_ emissions on ambient PM_2.5_ (step 2), as well as the accuracy of our mortality projections (step 3). There have been assessments of the performance of CAMx in North America and Europe that compare the model’s projections with readings at monitoring stations and with the results of other modeling platforms (Ferreira et al., 2012; Nopmongcol et al., 2012; Pirovano et al., 2012; Tesche et al., 2006), but none that we could find for India. The concentration-response functions we have used to estimate the mortality impacts of PM_2.5_ have been updated by the Global Burden of Disease (GBD) team (see Ostro et al., 2018 for a summary) and a recent reanalysis of prospective cohort data (Burnett et al., 2018) suggests much larger impacts of fine particles on mortality than those embodied in our analyses. It is also the case that we use national death rates to estimate mortality impacts, rather than state- (or district-) specific ones, which introduces additional error into the analysis.

Regarding the valuation of mortality impacts (step 4) we have used sensitivity analysis to examine the impact of alternate valuation assumptions, specifically, using different approaches to transfer the VSL from other countries to India and using different discount rates. As Tables 2 and 4 demonstrate, the magnitude of the present value of mortality benefits is very sensitive to both the VSL and the discount rate, as is the present value of mortality benefits net of scrubbing costs. We emphasize, however, that the ranking of locations based on net mortality benefits is unaffected by the choice of the VSLs or discount rates that we have considered. Because the morbidity benefits of scrubbing should be positively correlated with deaths avoided, this ranking should also be robust to the inclusion of morbidity benefits, which we have not quantified. Thus, our analysis can provide useful information for prioritizing locations for retrofitting plants with FGDs based on health considerations.

We emphasize, however, that in locations where the net mortality benefits of scrubbing are negative (as reported in Table 4) the total net benefits of scrubbing are not necessarily negative. We have not quantified (or monetized) the morbidity benefits of reduced PM_2.5_ concentrations, nor have we examined ecosystem or aesthetic benefits. These remain a subject for future research.

## Footnotes

1 Coal-fired power plants were responsible for approximately 8 percent of all premature deaths attributable to PM_2.5_ in India in 2015 and are projected to be responsible for 13 percent of PM_2.5_ deaths in 2050, under a scenario (S2) which assumes power plants become more energy-efficient. In a business-as-usual scenario, power plants would account for 23 percent of PM_2.5_ deaths in 2050 (GBD MAPS, 2018, Table 8).

2 Coal-fired power plants accounted for 76 percent of electricity generation and 59 percent of generating capacity in India in fiscal 2016 (CEA, 2017).

3 Their analysis covers 90 percent of coal-fired generating capacity in operation as of March 31, 2009.

4 A recent analysis by CSTEP (2018) of the costs and benefits of meeting the 2015 standards for SO_2_, NO*_x_* and PM likewise concludes that these emission standards led to benefits that exceed costs.

5 The 2015 regulations limit SO_2_ emissions to 200 mg Nm^−3^ for units installed before 2016 and 100 200 mg Nm^−3^ for units installed at the beginning of 2017. These standards will require the installation of FGDs.

6 Although Cropper et al. (2017) and CSTEP (2018) quantify the benefits of retrofitting scrubbers they do not emphasize how net benefits vary with plant location.

7 CSTEP (2018) reports that in 2015, 12 percent of coal burned by thermal power plants was imported. On average, imported coal, especially coal imported from Indonesia, has higher sulfur content. This implies that our estimates of the benefits of scrubbing may underestimate benefits.

8 http://www.camx.com.

9 It may, however, be possible to use a reduced-form model to estimate the impact of SO_2_ on fine particles, since sulfate concentrations are roughly linear in emissions, conditional on meteorological conditions. See for example, Zhou et al. (2006).

10 The meteorological data (wind, temperature, pressure, relative humidity, and precipitation) used in running the model were derived from the National Center for Environmental Prediction (NCEP)’s global reanalysis database and processed through the Weather Research and Forecasting (WRF) meteorological model at a one-hour temporal resolution. See also (Guttikunda & Jawahar, 2014, 2018).

11 Our population data are not stratified by age and sex. We use the national distribution of death rates by age and sex in calculating the mortality impacts of changes in fine particles attributed to each model plant.

12 See http://ghdx.healthdata.org/record/global-burden-disease-study-2010-gbd-2010-ambient-air-pollution-risk-model-1990-2010. An alternate set of concentration-response functions, reported in Burnett et al. (2018), do not require such detailed, disease-specific information on causes of death.

13 Specifically, we evaluated the change in relative risk at the population-weighted ambient concentration of PM_2.5_ within a 100 kilometer radius surrounding each plant, computed using the supplementary material from Brauer et al. (2012). Concentrations ranged from 15 to 46 μg m^−3^, with a mean of 27 μg m^−3^.

14 We use death rates by age and cause reported in the 2013 Global Burden of Disease. https://cloud.ihme.washington.edu/index.php/s/b89390325f728bbd99de0356d3be6900?path=%2FIHME%20GBD%202013%20Deaths%20by%20Cause%201990-2013.

15 We assume in our calculations of the health impacts of PM_2.5_ that each pollutant species (e.g., ammonium sulfate and ammonium nitrate) is equally toxic.

16 This is a conservative assumption. CSTEP in their analysis of the benefits and costs of alternate pollution control options for coal-fired power plants in India (CSTEP, 2018) assume that a wet limestone FGD removes 95 percent of SO_2_ emissions while a seawater FGD removes 92 percent.

17 The present value of premature deaths reduced assuming a 3 percent discount rate and the US EPA (2011) lag structure is 0.877 times the undiscounted number of deaths reduced. Using the five-year lag structure in EPA (2013) and a discount rate of 3 percent the present value of premature deaths reduced is 0.950 times the undiscounted number of premature deaths reduced. Using a discount rate of 8 percent, these numbers become 0.740 and 0.879. We conservatively assume that the present value of deaths reduced is 0.8 times the undiscounted number of deaths reduced.

18 Appendix Tables A1–A5 present benefits and net benefits in PPP dollars and also in rupees.

19 The IMF forecasts the rate of GDP growth in India to be 7.3 percent for 2018 and 7.5 percent for 2019. https://www.imf.org/en/Publications/WEO/Issues/2018/07/02/world-economic-outlook-update-july-2018. This implies a rate of per capita GDP growth of about 6.3 percent, assuming a rate of population growth of one percent.

20 The average age of persons over age 15 in India in 2015 was 43.8. Remaining life expectancy in the 40–45 age bracket was 34.4 years.

21 The U.S. EPA’s AP-42 database indicates that a wet scrubber can achieve up to 95 percent SO_2_ removal; equipment suppliers claim SO_2_ removal efficiencies of up to 99 percent with additives in the flue-gas stream.

22 FGDs are also planned for Bongaigaon, Mundra, Ratnagiri and Vindhyachal (Cropper et al., 2017).

23 The capital costs for a wFGD at the Dadri plant (https://www.businesstoday.in/current/economy-politics/bhel-bags-rs-560-crore-order-ntpc-install-emission-control-equipment-dadri-power-station/story/270662.html) in 2018 Rs. are similar to those for a wFGD at the Bongaigaon plant cited in Cropper et al. (2017) in 2013 Rs. We take this as evidence that the price in current Rs. has remained approximately constant over this period and convert the cost (0.60 crore/MW) to 2015 USD using the 2015 exchange rate (Rs. 64 = $1). The only data we have for swFGD costs are from Dahanu and Trombay. The cost of a swFGD on two 250 MW units at Dahanu (Rs. 232 crore) is more than twice the cost of the swFGD on a 250 MW unit at Trombay (Rs. 52 crore). We conservatively use the Dahanu figure, which implies a cost of 0.464 crore/MW. We convert this to 2015 dollars using the 2015 exchange rate.

24 The operating and maintenance cost assumptions and the capital cost assumptions used here are identical to those in Cropper et al. (2017) in Rs.; however, they are converted to 2013$ in Cropper et al. (2017) and to 2015$ here. The lower figures in the present paper reflect a change in the rupee–dollar exchange rate.

25 Operating and maintenance costs may increase due to increases in the cost of labor, limestone reagent, or increases in the cost of auxiliary power.

26 Per capita GDP, measured in 2014–15, has been converted to 2017$.

## Appendix A

**Table A1:** Present value of mortality benefits of an FGD in millions (2015 PPP$).

Plant name	VSL	$84,036	$160,000	$256,000	$256,000	$256,000
	r	3%	3%	3%	8%	12%
Dadri		5440	7190	11500	6950	5000
Unchahar		3570	4720	7550	4570	3280
Bakreswar		1630	2150	3440	2080	1500
Dahnu		905	1200	1910	1160	831
Talcher		698	923	1480	893	641
Koradi		480	635	1020	614	441
Rayalaseema		264	349	558	337	242
Tuticorin		194	256	410	248	178

**Table A2:** Net benefits from scrubbing in millions (2015 PPP$).

Plant name	VSL	$84,036	$160,000	$256,000	$256,000	$256,000
	r	3%	3%	3%	8%	12%
Dadri		4810	6550	10,900	6480	4580
Unchahar		2940	4090	6930	4090	2860
Bakreswar		1000	1530	2820	1600	1080
Dahnu		406	697	1410	773	500
Talcher		72	297	852	409	222
Koradi		–146	8.52	390	131	21.8
Rayalaseema		–362	–278	–68.2	–146	–177
Tuticorin		–305	–243	–89.4	–135	–153

**Table A3:** Present value of mortality benefits of an FGD in crores (2015 Rs.).

Plant name	VSL	$84,036	$160,000	$256,000	$256,000	$256,000
	r	3%	3%	3%	8%	12%
Dadri		9190	12,200	19,500	11,800	8440
Unchahar		6030	8000	12,700	7740	5540
Bakreswar		2750	3640	5820	3510	2530
Dahnu		1530	2020	3230	1950	1400
Talcher		1180	1560	2500	1510	1080
Koradi		811	1080	1720	1040	745
Rayalaseema		446	589	941	570	409
Tuticorin		328	433	691	419	301

**Table A4:** Operating characteristics for cost calculations: baseline assumptions.

		Benchmark	
Capacity utilization (%)		85	
Capital discount rate (%)		3%	
Plant life (retrofit) (yrs)		20	
		FGD type	
	Wet limestone		Seawater
Capital costs (crore Rs./MW)	0.600		0.46
Fixed operating costs (Rs./MWh)	25.0		19.0
Electricity costs (Rs./kWh)	3.50		3.50
Auxiliary consumption (%)	1.50		1.25
Annual FGD electricity cost for a 500 MW unit (crore Rs.)	19.5		16.3
PDV of costs for a retrofitted FGD (crore Rs.)	1060		845
PDV of costs for a new FGD (crore Rs.)	966		774

**Table A5:** Net Benefits from Scrubbing in Crores (2015 Rs.).s

Plant name	VSL r	$84,036 3%	$160,000 3%	$256,000 3%	$256,000 8%	$256,000 12%
Dadri		8130	11,100	18,400	10,900	7730
Unchahar		4980	6910	11,700	6910	4830
Bakreswar		1690	2580	4760	2700	1820
Dahnu		685	1180	2390	1310	845
Talcher		122	501	1440	691	375
Koradi		–247	14.4	659	221	36.8
Rayalaseema		–612	–469	–115	–246	–299
Tuticorin		–516	–411	–151	–228	–259
